# Insulin Versus Established GLP-1 Receptor Agonists, DPP-4 Inhibitors, and SGLT-2 Inhibitors for Uncontrolled Type 2 Diabetes Mellitus: A Systematic Review and Meta-Analysis of Randomized Controlled Trials

**DOI:** 10.7759/cureus.92175

**Published:** 2025-09-12

**Authors:** Ammar Ahmed, Zi Tan, Waddah Abd El-Radi, Krishnakumar Rajamani

**Affiliations:** 1 Department of Medicine, University of Minnesota School of Medicine, Minneapolis, USA; 2 Department of Endocrinology, Medical University of South Carolina (MUSC) Health Endocrinology at Nexton Medical Park, Summerville, USA; 3 Department of Internal Medicine, Rochester Regional Health, Rochester, USA; 4 Department of Medicine/Diabetes Endocrinology and Metabolism, Rochester Regional Health, Rochester, USA

**Keywords:** dpp-4 inhibitors, glp-1 receptor agonists, insulin, meta-analysis, non-insulin diabetes medications, sglt-2 inhibitors, systematic review, type 2 diabetes mellitus

## Abstract

Limited head-to-head studies compare established glucagon-like peptide-1 receptor agonists (GLP-1 RAs), dipeptidyl peptidase-4 inhibitors (DPP-4is), and sodium-glucose co-transporter-2 inhibitors (SGLT-2is) to insulin in the management of uncontrolled type 2 diabetes mellitus (T2DM). This systematic review and meta-analysis evaluated the efficacy and safety of traditional GLP-1 RAs, DPP-4is, and SGLT-2is compared with insulin. Comprehensive searches were conducted in the Cochrane Database, PubMed, MEDLINE, ClinicalTrials.gov, and EMBASE for publications from January 2010 to June 2022, with an additional search extended through June 2025 to capture newly published studies. Randomized controlled trials (RCTs) comparing insulin with established GLP-1 RAs, DPP-4is, or SGLT-2is were included. Thirteen trials involving 5,807 participants were identified. Nine trials compared GLP-1 RAs to insulin, four compared DPP-4is to insulin, and one examined SGLT-2i combined with DPP-4i versus insulin. Compared with insulin, traditional non-insulin agents were associated with greater reductions in hemoglobin A1c (HbA1c) (mean difference (MD) = -0.27, 95% confidence interval (CI) -0.5 to -0.03), body weight (MD = -3.27, 95% CI -4.16 to -2.38), and systolic blood pressure (MD = -3.55, 95% CI -4.92 to -2.17). Insulin use carried a higher relative risk of hypoglycemia (risk ratio (RR) = 2.24, 95% CI 1.88-2.67). Subgroup analyses showed that GLP-1 RAs were superior to insulin in reducing HbA1c and hypoglycemic events, whereas DPP-4is achieved similar glycemic control with improved safety profiles. These findings suggest that established GLP-1 RAs, DPP-4is, and SGLT-2is offer superior or comparable efficacy with better safety than insulin in insulin-naïve patients with uncontrolled T2DM.

## Introduction and background

Diabetes mellitus (DM) affects more than 38.4 million people in the United States, or approximately 11.6% of the US population [1,2]. Type 2 diabetes mellitus (T2DM) accounts for 90% to 95% of the population with DM [1,2]. T2DM is associated with a relative or complete impairment in insulin secretion, along with varying degrees of peripheral resistance to insulin action [3]. The beta-cell function is typically half that of healthy individuals at the time of diagnosis [3]. With disease progression, insulin output declines further, and many patients ultimately require treatment with exogenous insulin to maintain glycemic control [4]. Although insulin is highly efficacious, it can have severe side effects. Hypoglycemia and weight gain frequently complicate both the initiation and optimization of insulin therapy [4,5]. The leading cause of morbidity and mortality in patients with DM remains cardiovascular (CV) disease, which is estimated to be two to four times more common in patients with DM [6,7]. The risk of developing an adverse CV event in patients with an HbA1c ≥ 8% is 16% higher than in patients with an HbA1c between 6% and 8% [8].

The older antidiabetic agents, including metformin, thiazolidinediones (TZDs), sulfonylureas (SUs), meglitinides, and insulin, have been in use for years to manage T2DM [9-11]. The newer classes of medications, including dipeptidyl peptidase-4 inhibitors (DPP-4is), glucagon-like peptide-1 receptor agonists (GLP-1 RAs), and sodium-glucose cotransporter-2 inhibitors (SGLT-2is), have become increasingly common over the last few years due to their CV and renal protective effects [12]. GLP-1 RAs work by stimulating the release of endogenous insulin in the presence of elevated blood glucose (BG) levels, thereby reducing both fasting and postprandial BG levels [5]. GLP-1 RAs can also promote weight loss by causing early satiety [13,14]. DPP-4is modulate fasting BG, postprandial BG, and HbA1c levels by decreasing the inactivation of incretins such as GLP-1 and glucose-dependent insulinotropic polypeptide. This stimulates the release of insulin in a glucose-dependent manner [13,15]. SGLT-2is inhibit the reabsorption of glucose from the proximal tubule of the kidney, causing glycosuria [16].

In one open-label cohort study, sitagliptin (DPP-4i) was found to reduce HbA1c by 3% from a baseline HbA1c of 11.8% [17]. Similarly, a 2% reduction in HbA1c from a baseline of 9.1% was seen with dapagliflozin (SGLT-2i) [18]. Several studies have compared basal insulin to GLP-1 RAs [19]. One such study showed that exenatide use was associated with a greater reduction in HbA1c compared to insulin glargine in patients with a mean baseline HbA1c of ≥ 9.0% [20].

The therapeutic landscape for T2DM has continued to evolve, with newer agents, such as dual incretin receptor agonists (e.g., tirzepatide), showing promising results in recent clinical trials. However, the established glucose-lowering agents, including GLP-1 RAs (exenatide, liraglutide, dulaglutide, semaglutide), DPP-4is (sitagliptin, saxagliptin, linagliptin, alogliptin), and SGLT-2is (canagliflozin, dapagliflozin, empagliflozin, ertugliflozin), remain the most widely available and prescribed non-insulin therapies globally. Understanding their comparative effectiveness against insulin remains crucial for clinical decision-making, particularly in healthcare settings where newer agents may not be accessible due to cost or availability constraints.

Every few years, the diabetes community reevaluates and updates the current recommendations for T2DM treatment to reflect new information from clinical research and practice. The current guidelines of the American Association of Clinical Endocrinologists (AACE) and the American Diabetes Association (ADA) recommend considering the initiation of insulin for patients with T2DM who have HbA1c levels exceeding 9.0% or 10%, respectively [21,22]. However, these recommendations are based on expert opinion rather than randomized controlled trials (RCTs). The 2025 ADA Standards of Care emphasize individualized pharmacologic approaches that address both glycemic and weight goals, with a preferential use of agents that reduce the risk of CV and kidney disease [23]. The 2022 ADA/European Association for the Study of Diabetes (EASD) consensus report advocates for a holistic, person-centered approach that considers weight management as integral to diabetes care, with specific recommendations for established and newer glucose-lowering medications [24]. Given the efficacy and additional benefits associated with the use of GLP-1 RAs, DPP-4is, and SGLT-2is, should these medications be the initial preferred treatments for patients with an HbA1c > 9% over insulin?

Several trials and reviews have compared individual GLP-1 RAs, DPP-4is, or SGLT-2is to insulin in poorly controlled T2DM patients. However, these studies typically evaluated single agents rather than examining the broader class effects of established non-insulin therapies. To our knowledge, no comprehensive meta-analysis has systematically evaluated these established glucose-lowering medications as distinct classes compared to insulin. Therefore, we conducted this systematic review and meta-analysis to compare the efficacy and safety of traditional GLP-1 RAs, DPP-4is, and SGLT-2is to insulin in poorly controlled T2DM, providing evidence to guide clinical practice with widely available therapeutic options. Our secondary aims were to investigate the effects of these medications on body weight and systolic blood pressure (SBP).

## Review

Methodology

Information Sources and Search Strategy

All published trials from January 2010 to June 2022 were searched on PubMed, MEDLINE, Embase, the Cochrane Central Register of Controlled Trials, and ClinicalTrials.gov. A secondary comprehensive search was conducted from June 2022 to June 2025 to capture any newly published studies. The search focused on established non-insulin diabetes medications available during this period, including traditional GLP-1 RAs (exenatide, liraglutide, dulaglutide, semaglutide, lixisenatide, albiglutide, taspoglutide), DPP-4is (sitagliptin, saxagliptin, linagliptin, alogliptin, vildagliptin), and SGLT-2is (canagliflozin, dapagliflozin, empagliflozin, ertugliflozin). Dual incretin receptor agonists (such as tirzepatide) were excluded because they represent a mechanistically distinct class that warrants separate analysis.

The search strategy combined both keywords and MeSH terms. The following terms were applied: (“T2DM”[MeSH] OR “T2DM” OR “type 2 diabetes”) AND (“insulin” OR “insulin therapy”[MeSH] OR insulin glargine OR insulin degludec OR insulin detemir OR NPH insulin OR basal insulin) AND (“GLP-1 receptor agonists”[MeSH] OR “GLP-1 receptor agonists” OR exenatide OR liraglutide OR dulaglutide OR semaglutide OR lixisenatide OR albiglutide OR taspoglutide) OR (“DPP-4 inhibitors”[MeSH] OR “DPP-4 inhibitors” OR sitagliptin OR saxagliptin OR linagliptin OR alogliptin OR vildagliptin) OR (“SGLT2 inhibitors”[MeSH] OR “SGLT2 inhibitors” OR canagliflozin OR dapagliflozin OR empagliflozin OR ertugliflozin).

Selection Criteria

According to our protocol's criteria, all RCTs published in English were included. All participants were adultswith T2DM with a baseline mean HbA1c ≥ 8% at the start of the trial. The included trials had an intervention duration of at least 24 weeks. All included trials reported HbA1c at the end of 24-30 weeks. Trials involving pregnant or breastfeeding participants were excluded. Comparisons were made between the first group (basal insulin, basal-bolus insulin regimens, and premixed insulins) and the second group (DPP-4is, GLP-1 RAs, or SGLT-2is). Trials that compared medications within the same class were excluded. In each comparison, background treatment was defined as the antidiabetic medications used in both the intervention and control groups after randomization. Eligible background therapy was either no background treatment or metformin-based background treatment (metformin only or metformin plus another antidiabetic medication).

After screening, two independent reviewers (AA, WA) screened titles and abstracts and examined full texts of potentially eligible trials. Disagreements were resolved through discussion with a third reviewer (ZT).

Data Extraction Outcomes and Quality Assessment

Data were extracted from the full texts of the published studies included. Two reviewers extracted the data independently. A pair of reviewers assessed risk of bias (WA, ZT); discrepancies were resolved with a third reviewer (AA). For each medication, outcome data were merged from all approved doses into a single intervention group. If outcomes were reported at multiple time points, results were extracted at 24-30 weeks or six months. The change from baseline HbA1c was considered the primary outcome. Secondary outcomes were hypoglycemic events, body weight change, and SBP change. Extracted data included participant numbers, demographics, trial duration, and the type of antidiabetic medication used. Additional data included the mean change in HbA1c (SD), the mean change in body weight (SD), and the frequency of hypoglycemic episodes.

Data Synthesis and Analysis

The efficacy of non-insulin agents versus insulin was compared in the included RCTs. Outcomes compared included HbA1c change, hypoglycemic events, body weight change, and SBP change at 24-30 weeks. HbA1c, SBP, and body weight were analyzed as continuous variables and reported as absolute mean differences. Safety was assessed by risk of hypoglycemia, analyzed as a dichotomous variable, and expressed as a risk ratio. Data were analyzed using a random-effects model with the inverse variance method. Heterogeneity was assessed with chi-square and tau-square tests. All analyses were conducted in RevMan (version 5.4).

Results

Study Collection and Characteristics

**Figure 1:**
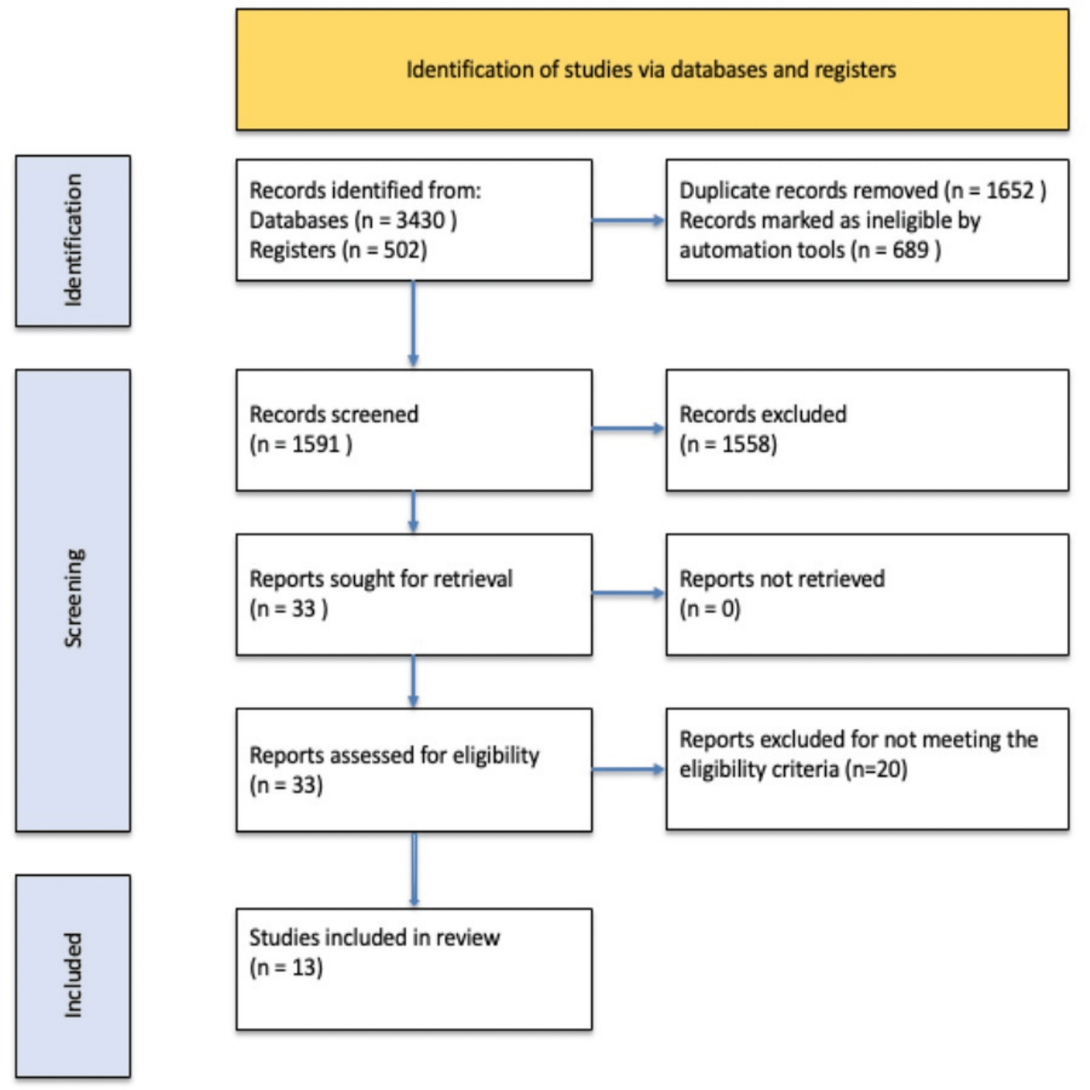
PRISMA 2020 flow diagram showing the selection process for included studies. Out of 3,932 records identified, 13 studies met eligibility criteria and were included in the review. PRISMA, Preferred Reporting Items for Systematic Reviews and Meta-Analyses

A total of 3,932 clinical trials were retrieved from the initial search (Figure 1). The secondary search (June 2022-June 2025) identified an additional 1,247 records, none of which met the inclusion criteria. In total, 3,899 articles were excluded based on title/abstract. Thirty-three trials underwent full-text screening. Thirteen RCTs (5,807 participants) [19,22,25-35] met all inclusion criteria and were included in the meta-analysis [19,22,25-35]. Twenty trials were excluded. Nine RCTs compared GLP-1 RAs with insulin in inadequately controlled T2DM [19,22,25,27-30,32,35]. Four RCTs compared DPP-4is with basal insulin [26,31,33,34]. One RCT examined SGLT-2i + DPP-4i versus insulin [34].

The design and baseline characteristics of the included studies are presented in Table 1. All RCTs reported mean HbA1c and hypoglycemia outcomes; not all reported every secondary outcome. Seven GLP-1 RA RCTs [19,25,27-30,35] and three DPP-4i RCTs [26,31,33] reported adequate data on weight change. Three GLP-1 RA RCTs reported SBP changes [25,27,35].

**Table 1:** Characteristics of included RCTs comparing GLP-1 receptor agonists or DPP-4 inhibitors with basal insulin in patients with T2DM. This table summarizes the design and baseline characteristics of the 13 RCTs included in this study. Each study is cited with its reference number in square brackets. Reported data include trial setting, duration of follow-up, number of participants, baseline age, HbA1c, and BMI, as well as the intervention drug and comparator (basal insulin analogs), and the primary and key secondary outcomes. RCT, randomized controlled trial; T2DM, type 2 diabetes mellitus; SU, sulfonylurea; FPG, fasting plasma glucose; BMI, body mass index; GLP-1, glucagon-like peptide-1; DPP-4, dipeptidyl peptidase-4; CRP, C-reactive protein; BP, blood pressure; HbA1c, hemoglobin A1c

Study	Setting	Follow-up	Participants	Baseline data	Intervention	Outcomes
Abdul-Ghani et al. [22]	Single-center, open-label RCT	6 months	*N* = 101, poorly controlled T2DM on biguanides and/or SU	Age: 50 vs. 52 years; HbA1c: 11.5 vs. 11.2%; BMI: 31.3 vs. 29.7 kg/m^2^	Pioglitazone (30 mg/d) + Exenatide (2 mg/week); Comparator: Basal/bolus insulin	Primary: HbA1c change; secondary: FPG, weight change
Araki et al. [19]	Multicenter, open-label RCT	26 weeks	*N* = 361, poorly controlled T2DM on biguanides and/or SU	Age: 57.5 vs. 56.1 years; HbA1c: 8.1 vs. 8.0%; BMI: 26.1 vs. 25.9 kg/m^2^	Dulaglutide (0.75 mg/week); Comparator: Basal insulin (glargine)	Primary: HbA1c change; Secondary: FPG, weight change
Aroda et al. [25]	Multicenter, Open-label RCT	30 weeks	*N* = 1089, poorly controlled T2DM on biguanides and/or SU	Age: 56.7 vs. 56.2 years; HbA1c: 8.3 vs. 8.1%; BMI: 33 vs. 33 kg/m^2^	Semaglutide (1 mg/week); Comparator: Basal insulin (glargine)	Primary: HbA1c change; Secondary: FPG, weight/BMI, lipids, BP, CRP, PAI-1
Aschner et al. [26]	Multicenter, Open-label RCT	24 weeks	*N* = 515, poorly controlled T2DM on biguanides	Age: 53.3 vs. 53.9 years; HbA1c: 8.5 vs. 8.5%; BMI: 31.3 vs. 31.1 kg/m^2^	Sitagliptin (100 mg/d); Comparator: Basal insulin (glargine)	Primary: HbA1c change; Secondary: FPG, weight, lipid profile
D’Alessio et al. [27]	Multicenter, Open-label RCT	24 weeks	*N* = 944, poorly controlled T2DM on biguanides and/or SU	Age: 57.4 vs. 57.1 years; HbA1c: 9.1 vs. 9.0%; BMI: 31.8 vs. 32.0 kg/m^2^	Liraglutide (0.6–1.8 mg/d); Comparator: Basal insulin (glargine)	Primary: % achieving HbA1c ≤7.0%; Secondary: HbA1c change, weight change
Davies et al. [28]	Multicenter, Open-label RCT	26 weeks	*N* = 216, poorly controlled T2DM on biguanides and/or SU	HbA1c: 8.37 vs. 8.35%; BMI: 33.7 vs. 33.7 kg/m^2^	Exenatide (2 mg/week); Comparator: Basal insulin (detemir)	Primary: HbA1c ≤7.0% + weight loss ≥1 kg; Secondary: FPG, HbA1c, weight, lipids, CRP
Inagaki et al. [30]	Multicenter, Open-label RCT	26 weeks	*N* = 420, poorly controlled T2DM on biguanides and/or SU	Age: 57.1 vs. 56.4 years; HbA1c: 8.5 vs. 8.5%; BMI: 26.1 vs. 26.2 kg/m^2^	Exenatide (2 mg/week); Comparator: Basal insulin (glargine)	Primary: HbA1c change; Secondary: % achieving HbA1c ≤7.0%/≤6.5%, weight change
Grimm et al. [29]	Multicenter, Open-label RCT	26 weeks	*N* = 456, poorly controlled T2DM on biguanides and/or SU	Age: 58 vs. 58 years; HbA1c: 8.3 vs. 8.3%; BMI: 32.3 vs. 32.3 kg/m^2^	Exenatide (2 mg/week); Comparator: Basal insulin (glargine)	Primary: HbA1c, weight change; Secondary: % achieving HbA1c <7%, hypoglycemia
Ji et al. [31]	Multicenter, Open-label RCT	24 weeks	*N* = 166, newly diagnosed T2DM	Age: 54.1 vs. 53.4 years; HbA1c: 9.8 vs. 9.7%; BMI: 27.2 vs. 29.0 kg/m^2^	Sitagliptin (50 mg/d) + Metformin; Comparator: Basal insulin (glargine) + Metformin	Primary: HbA1c change; Secondary: BMI, lipid profile
Nauck et al. [32]	Multicenter, Open-label RCT	24 weeks	*N* = 1094, poorly controlled T2DM on biguanides and/or SU	Age: 57 vs. 58 years; HbA1c: 8.3 vs. 8.4%; BMI: 32.4 vs. 32.7 kg/m^2^	Taspoglutide (20 mg/week); Comparator: Basal insulin (glargine)	Primary: HbA1c change; Secondary: % achieving HbA1c <7%, weight, lipids, CRP, SBP
Philis-Tsimikas et al. [33]	Multicenter, Open-label RCT	26 weeks	*N* = 458, poorly controlled T2DM on ≥1 OAD (biguanides, SU, glinides, pioglitazone)	Age: 54.9 vs. 56.4 years; HbA1c: 9.0 vs. 8.8%; BMI: 30.8 vs. 30.0 kg/m^2^	Sitagliptin (100 mg/d); Comparator: Basal insulin (degludec)	Primary: HbA1c change; Secondary: FPG, % achieving HbA1c <7%
Vilsbøll et al. [34]	Multicenter, Open-label RCT	24 weeks	*N* = 643, poorly controlled T2DM on biguanides and/or SU	Age: 55.7 vs. 55.3 years; HbA1c: 9.0 vs. 9.1%; BMI: 32.5 vs. 32.0 kg/m^2^	Saxagliptin (5 mg/d) + Dapagliflozin (10 mg/d); Comparator: Basal insulin (glargine)	Primary: HbA1c change; Secondary: weight change
Wang et al. [35]	Multicenter, Open-label RCT	26 & 52 weeks	*N* = 774, poorly controlled T2DM on biguanides and/or SU	Age: 55 vs. 55.4 years; HbA1c: 8.5 vs. 8.3%; BMI: 26.6 vs. 26.7 kg/m^2^	Dulaglutide (1.5 mg/week); Comparator: Basal insulin (glargine)	Primary: HbA1c change at 26 weeks; Secondary: HbA1c change at 52 weeks

Risk of Bias Assessment

**Figure 2:**
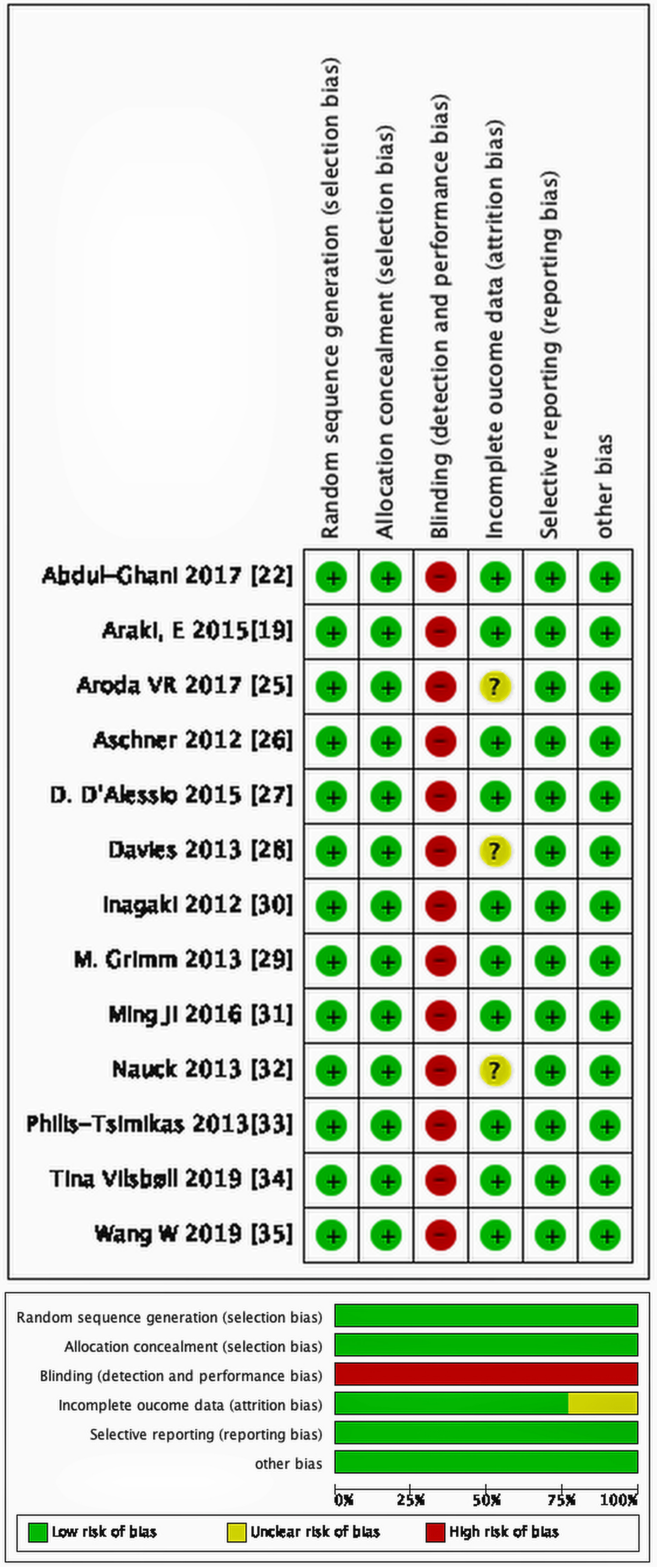
Risk of bias assessment of included randomized controlled trials. Risk of bias assessment for the included studies using the Cochrane Risk of Bias tool. Each domain is categorized as low risk (green), unclear risk (yellow), or high risk (red). The upper panel shows the risk of bias judgment for each individual study, while the lower panel summarizes the proportion of studies with each risk level across all domains.

The quality of the included studies in this review was evaluated by assessing the risk of bias using the Cochrane Collaboration's tool [36]. The items assessed were sequence generation, allocation concealment, blinding of outcome assessor, incomplete outcome data, and selective outcome reporting. The risk of bias was graded as unclear, high, or low risk. Most of the included studies were of high quality with a low risk of bias. None of the included trials were blinded; therefore, the risk of detection and performance bias was high. Three of the studies had incomplete data; thus, the risk of attrition bias could not be assessed. Details of the risk of bias assessment are shown in Figure 2. All the included studies were funded by pharmaceutical companies.

Statistical Analysis

**Figure 3:**
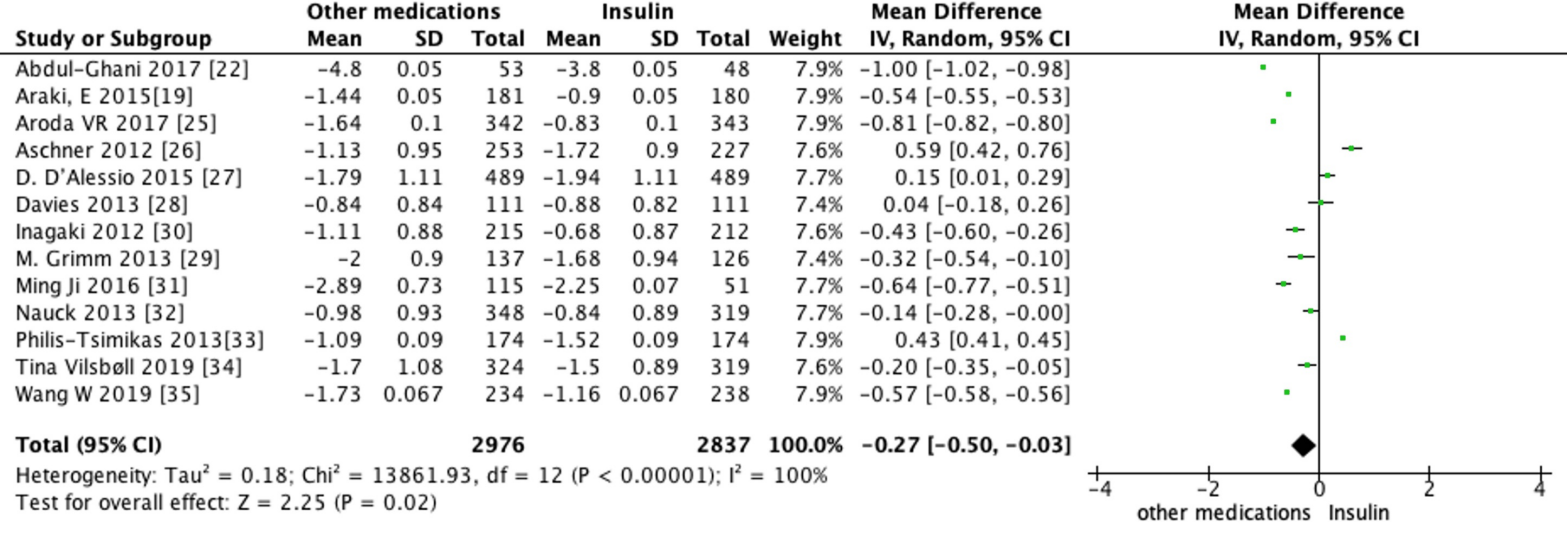
Pooled analysis of the mean difference (MD) in change in HbA1c between non-insulin agents and insulin (random-effects model). Forest plot showing the pooled MD in change in HbA1c between established non-insulin antidiabetic medications and insulin, analyzed using a random-effects model. Each square represents an individual study with its weight, and horizontal lines denote 95% confidence intervals (CIs). The diamond indicates the overall pooled effect estimate (MD = -0.27%, 95% CI -0.50 to -0.03, *P *= 0.02). SD, standard deviation

**Figure 4:**
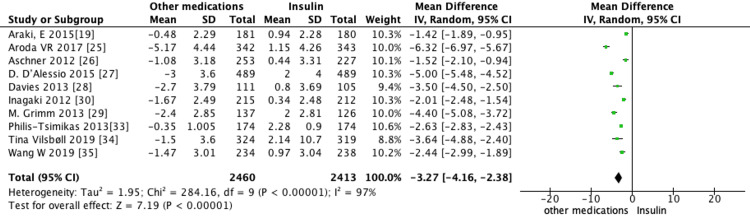
Pooled analysis of mean difference (MD) in body weight change between non-insulin agents and insulin (random-effects model). Forest plot showing the pooled MD in body weight change (kg) between established non-insulin antidiabetic agents and insulin. Each study is displayed with its effect estimate and 95% confidence interval (CI), while the diamond represents the overall pooled effect (MD = -3.27 kg, 95% CI -4.16 to -2.38, *P* < 0.00001). SD, standard deviation

**Figure 5:**
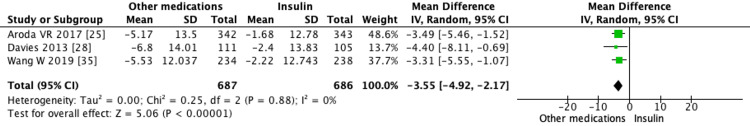
Pooled analysis of mean difference (MD) in systolic blood pressure change between non-insulin agents and insulin (random-effects model). Forest plot showing the pooled MD in systolic blood pressure (mmHg) change between established non-insulin antidiabetic agents and insulin, analyzed using a random-effects model. Each study’s effect estimate with 95% confidence interval (CI) is shown, and the diamond represents the overall pooled effect (MD = -3.55 mmHg, 95% CI -4.92 to -2.17, *P* < 0.00001). SD, standard deviation

**Figure 6:**
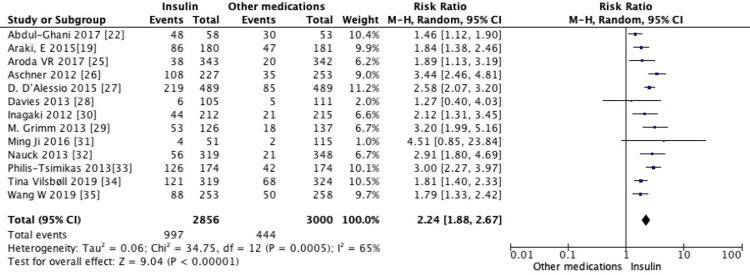
Pooled analysis of risk ratio for hypoglycemic events with non-insulin agents versus insulin (random-effects model). Forest plot showing the pooled risk ratio (RR) of hypoglycemic events comparing established non-insulin antidiabetic agents with insulin, analyzed using a random-effects model. Individual study estimates with 95% confidence intervals (CIs) are shown, and the diamond represents the overall pooled effect (RR = 2.24, 95% CI 1.88-2.67, *P* < 0.00001), indicating a higher risk of hypoglycemia with insulin.

Pooled analysis of all 13 trials revealed the use of non-insulin diabetes medications led to a significant reduction in mean HbA1c (MD = -0.27, 95% CI -0.5 to -0.03, *P* = 0.02) (Figure 3), weight (MD = -3.27, 95% CI -4.16 to -2.38, *P *< 0.00001) (Figure 4), and SBP (MD = -3.55, 95% CI -4.92 to -2.17, *P *< 0.00001) (Figure 5) when compared to the use of insulin. Furthermore, insulin use was associated with a higher incidence of hypoglycemic events compared to non-insulin diabetes medications (RR = 2.24, 95% CI 1.88-2.67, *P* < 0.00001) (Figure 6).

Subgroup Analysis

**Figure 7:**
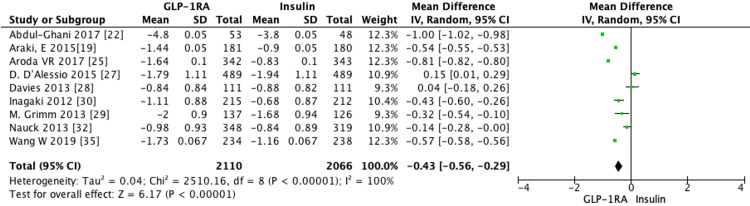
Subgroup analysis of the mean difference (MD) in change in HbA1c between GLP-1 receptor agonists and insulin (random-effects model). Forest plot showing the MD in change in HbA1c (%) between GLP-1 receptor agonists and insulin, analyzed using a random-effects model. Individual study estimates are shown with 95% confidence intervals (CIs), and the diamond represents the overall pooled effect (MD = -0.43%, 95% CI -0.56 to -0.29, *P* < 0.00001). SD, standard deviation

GLP-1 RA trials: Nine RCTs involving GLP-1 RAs (*N* = 4,176 participants) showed a statistically significant decrease in mean HbA1c from baseline compared to insulin (MD = -0.43, 95% CI -0.56 to -0.29, *P* < 0.00001) (Figure 7). One trial comparing the combination of GLP-1 RA and TZD to basal insulin achieved the most significant reduction in HbA1c of 1% (*P* < 0.001) [19].

Several different GLP-1 RAs were used in these RCTs. Exenatide 2 mg weekly was administered in four RCTs [19,27-29]. Dulaglutide 0.75 mg [22] and 1.5 mg [35] were used weekly in two separate RCTs. Additionally, three RCTs included semaglutide 1 mg weekly [25], taspoglutide 20 mg weekly [32], and liraglutide 0.6-1.8 mg daily [28].

**Figure 8:**
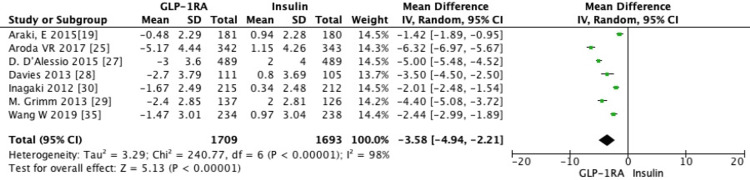
Subgroup analysis of the mean difference (MD) in weight change between GLP-1 receptor agonists and insulin (random-effects model). Forest plot showing the pooled MD in weight change (kg) between GLP-1 receptor agonists and insulin, analyzed using a random-effects model. Each square represents an individual study estimate with 95% confidence intervals (CIs), and the diamond represents the overall pooled effect (MD = -3.58%, 95% CI -4.94 to -2.21, *P* < 0.00001), favoring GLP-1 receptor agonists. SD, standard deviation

Seven RCTs evaluated the mean difference in weight change between GLP-1 RAs and insulin [22,25,27-29,35]. Pooled analysis showed a statistically significant decrease in weight (MD = -3.58, 95% CI -4.94 to -2.21, *P *= 0.0001) with GLP-1 RAs compared to insulin (Figure 8).

**Figure 9:**
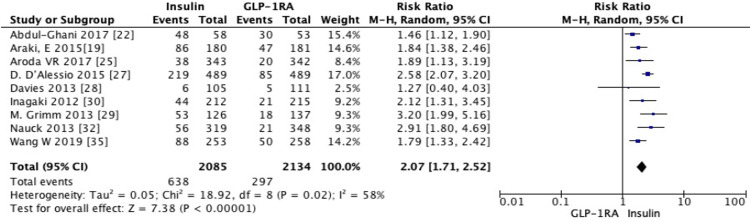
Subgroup analysis of risk ratio (RR) for hypoglycemic events with GLP-1 receptor agonists versus insulin (random-effects model). Forest plot showing the pooled RR of hypoglycemic events comparing GLP-1 receptor agonists with insulin, analyzed using a random-effects model. Individual study estimates with 95% confidence intervals (CIs) are shown, and the diamond indicates the overall pooled effect (RR = 2.07, 95% CI 1.71-2.52, *P* < 0.00001), demonstrating a higher risk of hypoglycemia with insulin. SD, standard deviation; GLP-1, glucagon-like peptide-1

There was also a statistically significant increase in the risk of hypoglycemic events with insulin compared to GLP-1 RA (RR = 2.07, 95% CI 1.71-2.52, *P* < 0.00001) (Figure 9).

DPP-4i Trials

**Figure 10:**
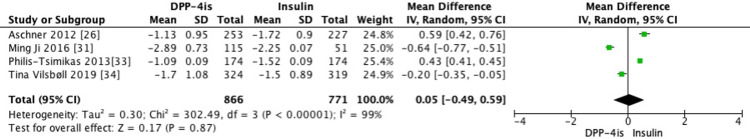
Subgroup analysis of the mean difference (MD) in change in HbA1c between DPP-4 inhibitors and insulin (random-effects model) Forest plot showing the pooled mean difference in change in HbA1c (%) between DPP-4 inhibitors and insulin, analyzed using a random-effects model. Each square represents the effect estimate of an individual study with its 95% confidence interval (CI), and the diamond indicates the overall pooled effect (MD = 0.05%, 95% CI -0.49 to 0.59, *P* = 0.87), showing no significant difference between groups.

**Figure 11:**
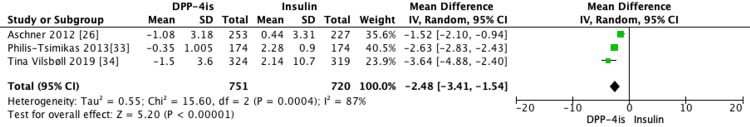
Subgroup analysis of the mean difference (MD) in weight change between DPP-4 inhibitors and insulin (random-effects model). Forest plot showing the pooled MD in body weight change (kg) between DPP-4 inhibitors and insulin, analyzed using a random-effects model. Individual study estimates are shown with 95% confidence intervals (CIs), and the diamond represents the overall pooled effect (MD = –2.48 kg, 95% CI -3.41 to -1.54, *P* < 0.00001), indicating greater weight reduction with DPP-4 inhibitors compared with insulin. DPP-4, dipeptidyl peptidase-4

Four RCTs compared the use of DPP-4i therapy with insulin alone [26,31,33,34]. Data for the secondary outcomes were not uniformly available within these four studies. Therefore, the mean changes in HbA1c, weight, and risk of hypoglycemic events from these studies were assessed. Compared to basal insulin, DPP-4is did not show a difference in reducing mean HbA1c from baseline (MD = 0.05, 95% CI -0.49 to 0.59, *P* = 0.87) (Figure 10). However, they were significantly associated with weight loss (MD = -2.48, 95% CI -3.41 to -1.54, *P* = 0.00001) (Figure 11).

**Figure 12:**
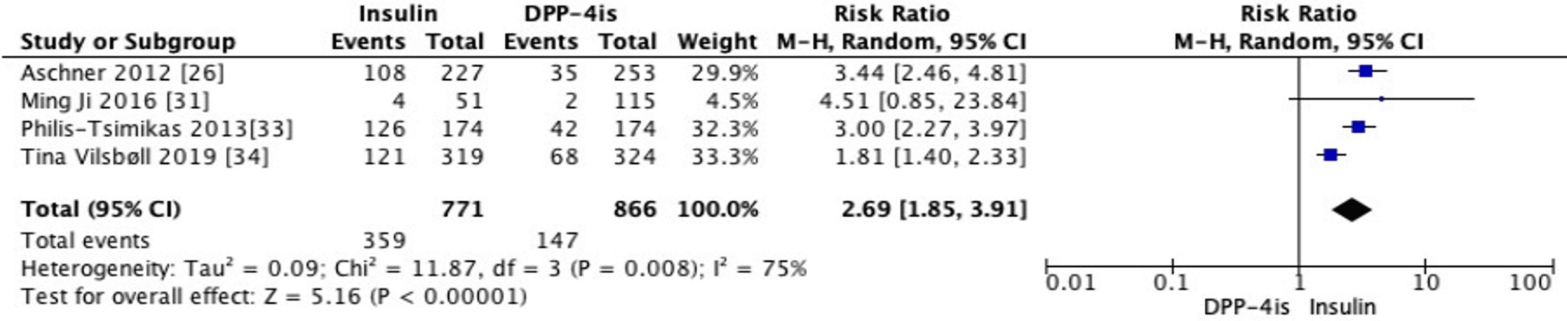
Subgroup analysis of risk ratio (RR) for hypoglycemic events with DPP-4 inhibitors versus insulin (random-effects model). Forest plot showing the pooled RR of hypoglycemic events comparing DPP-4 inhibitors with insulin, analyzed using a random-effects model. Each square represents an individual study estimate with its 95% confidence interval (CI), and the diamond indicates the overall pooled effect (RR = 2.69, 95% CI 1.85-3.91, *P* < 0.00001), demonstrating a higher risk of hypoglycemia with insulin. DPP-4, dipeptidyl peptidase-4

One RCT examined a DPP-4i combined with an SGLT-2i [34]. Insulin use was associated with a higher relative risk of hypoglycemic events compared to DPP-4is (RR = 2.69, 95% CI 1.85-3.91, *P* < 0.00001) (Figure 12).

Discussion

The initiation of insulin for poorly controlled diabetes has been the standard of treatment for several years; however, insulin has been associated with significant side effects, including hypoglycemia and weight gain. The development of novel diabetic therapies has opened the door for treatment options that offer glycemic control with additional benefits. This study aimed to further compare the efficacy of GLP-1 RAs, DPP-4is, and SGLT-2is to insulin in patients with T2DM and an HbA1c ≥ 8%. We found that the GLP-1 RAs and DPP-4is were best suited to meet our protocol's criteria; however, one trial utilizing a combination of an SGLT-2i and a DPP-4i met our analysis eligibility criteria. This analysis included nine GLP-1 RA trials involving the use of exenatide +/- pioglitazone, dulaglutide, liraglutide, taspoglutide, and semaglutide. Sitagliptin and saxagliptin were the only DPP-4i medications included in trials that met this analysis’s inclusion criteria. The majority of trials used basal insulin treatment alone, but one trial used both basal and prandial insulin [22]. A pooled analysis of all included trials showed a significant decrease in HbA1c with the use of non-insulin medications compared to insulin. In the subgroup analysis, the GLP-1 RAs displayed a greater effect in reducing HbA1c, weight, and SBP. The DPP-4is did not show any difference in reducing mean HbA1c but were associated with more weight loss compared to insulin alone.

A safety analysis was conducted by assessing the risk of hypoglycemia. A statistically significant reduction in the risk of hypoglycemia was observed with the use of GLP-1 RAs and DPP-4is compared to insulin. Subgroup analysis of GLP-1 RAs and DPP-4is revealed similar results when compared to insulin alone.

Contemporary Context and Clinical Relevance

Our findings remain highly relevant to current clinical practice, where traditional GLP-1 RAs, DPP-4is, and SGLT-2is constitute the majority of non-insulin prescriptions globally. While newer agents such as tirzepatide (a dual GLP-1/GIP receptor agonist) have demonstrated superior efficacy in recent trials, including SURPASS-3, SURPASS-4, and SURPASS-AP-Combo, these agents are not yet widely available in many healthcare systems due to cost and accessibility constraints. The established agents examined in our analysis represent the backbone of modern diabetes therapy and remain first-line considerations in current clinical guidelines.

The 2025 ADA Standards of Care emphasize that "pharmacologic therapies should address both individualized glycemic and weight goals in adults with type 2 diabetes" and prioritize "glycemic management agents that also reduce cardiovascular and kidney disease risk" [23]. The 2022 ADA/EASD consensus report emphasizes the importance of weight management as a vital component of diabetes care, recommending agents with proven cardiovascular and renal benefits for high-risk patients [24]. These guidelines support our findings demonstrating the superiority of established GLP-1 RAs and the favorable risk-benefit profile of DPP-4is compared to insulin.

This meta-analysis demonstrated robust and consistent findings supporting the benefit of using a GLP-1 RA in uncontrolled T2DM (Figures 1-2). Our findings are similar to a previous meta-analysis, which showed that GLP-1 RAs were more effective in reducing HbA1c, weight, and SBP with a lower risk of hypoglycemia when compared to insulin. However, this previous meta-analysis included 19 trials with a minimum follow-up duration of only 12 weeks. Furthermore, there was no minimum cut-off of the mean HbA1c [37]. Another meta-analysis showed similar results, but the interpretation of hypoglycemia risk was limited by inconsistent definitions and reporting of the hypoglycemic events [38]. The previous meta-analyses comparing DPP-4is to insulin showed no significant reduction in HbA1c between insulin and DPP-4is [39]. To our knowledge, no previous meta-analysis has been conducted comparing SGLT-2is to insulin in patients with uncontrolled T2DM.

Clinical Implications

These results support current clinical practice patterns, which favor the use of established non-insulin medications over insulin initiation in patients with uncontrolled T2DM. The combination of superior or equivalent glycemic control, weight reduction benefits, cardiovascular advantages (particularly with GLP-1 RAs and SGLT-2is), and a lower risk of hypoglycemia positions these established therapies as preferred second-line options after metformin failure. For DPP-4is, while glycemic efficacy was similar to insulin, the favorable safety profile and weight neutrality support their use in patients where GLP-1 RAs or SGLT-2is are contraindicated or not tolerated. The limited SGLT-2i representation in our analysis (only one combination trial [34]) reflects the research focus on cardiovascular and renal outcome studies rather than direct glycemic comparisons with insulin. However, these agents have demonstrated significant cardiorenal benefits in dedicated outcome trials and warrant consideration in appropriate patients.

The current guideline of the ADA suggests that early introduction of insulin should be considered in T2DM patients with HbA1c levels exceeding 10% or BG ≥ 300 mg/dL [40]. The current AACE guideline recommends considering the initiation of insulin for T2DM patients with HbA1c levels exceeding 9.0% [12]. The results of this study support the higher efficacy and safety of GLP-1 RAs compared to insulin in treating patients with T2DM and an HbA1c level of > 8%.

Limitations

One limitation of this meta-analysis is its focus on established non-insulin diabetes medications available as of June 2025. Newer dual incretin receptor agonists, particularly tirzepatide, have demonstrated superior efficacy to insulin in recent randomized controlled trials and represent a mechanistically distinct class of medications that warrant separate systematic evaluation. The limited number of SGLT-2i versus insulin head-to-head trials meeting our inclusion criteria reflects the research focus on cardiovascular and renal outcome studies rather than direct glycemic comparisons with insulin [34]. The unknown long-term durability of treatment effects remains a limitation, as included trial durations ranged from 24 to 30 weeks (mean 26.4 weeks). Additionally, all included studies were open-label trials, which may introduce potential detection and performance bias. The pharmaceutical industry funded all included trials, and several factors beyond efficacy and safety (comorbidities, age, lifestyle, cost) influence medication selection but could not be adequately assessed from available trials data.

Future research should specifically examine dual incretin agonists versus insulin, conduct additional head-to-head trials comparing SGLT-2is versus insulin, evaluate the longer-term durability of effects, and include real-world effectiveness studies to complement the randomized trial evidence.

## Conclusions

This systematic review and meta-analysis demonstrates that established non-insulin diabetes medications provide superior or equivalent glycemic control, with significant advantages in weight reduction, blood pressure control, and a reduced risk of hypoglycemia, compared to insulin in patients with poorly controlled T2DM. Traditional GLP-1 RAs demonstrated superior efficacy across multiple outcomes, while DPP-4is showed similar glycemic efficacy to insulin, with exceptional safety profiles. Although limited head-to-head data exist for SGLT-2is versus insulin, the available evidence supports their consideration alongside other established therapies. These findings support the preferential use of established non-insulin diabetes medications over insulin initiation in appropriate insulin-naïve patients with uncontrolled T2DM, providing evidence-based guidance for clinical decision-making with widely available therapeutic options that align with current ADA and ADA/EASD recommendations emphasizing individualized, person-centered care.
